# Referral for Weight Management in Early Intervention in Psychosis

**DOI:** 10.1192/bjo.2025.10561

**Published:** 2025-06-20

**Authors:** Richard Chater, Priyanka Kaushal

**Affiliations:** Forward Thinking Birmingham, Birmingham, United Kingdom

## Abstract

**Aims:** People with psychosis die, on average, 15–20 years earlier than the general population, with cardio-metabolic syndrome as the biggest contributor to excess mortality. Compounding this, poor concordance with antipsychotic medications is often attributed to weight gain.

In recent years, there has been rising media coverage, public awareness, and utilisation of weight loss medications such as Ozempic (semaglutide). NICE Weight Management guidelines recommend diet and lifestyle advice, followed by escalation to pharmacological management or specialist weight management services for those with a BMI over 30, or a BMI over 28 with other risk factors for cardio-metabolic syndrome. Furthermore, a 2022 systematic review showed pharmacological weight loss management to be effective in those with psychosis.

With this audit, we sought to determine the degree to which a Birmingham Early Intervention in Psychosis service was adhering to the steps laid out in the NICE Weight management guideline (NG246) during annual physical health assessments.

**Methods:** We assessed the documentation of all physical health assessments completed by the East Birmingham Early Intervention in Psychosis Service between August and November 2024. We then excluded any patients with a BMI under 30, providing a sample of 17 patients who would fit the criteria of the NICE Weight Management guideline. We then determined if they had previously received diet and exercise advice, if they or the clinician were concerned about their BMI, and finally if the clinician had documented advice to seek weight management support from their GP.

**Results:** We found that of the 17 patients with a BMI over 30, in 12 cases (71%) the patient or clinician had recorded concerns about their weight. Sixteen (94%) had been given diet and exercise advice in their most recent physical health review. Of the 12 (71%) for which concern had been documented, 6 (50%) had previously received diet and exercise advice at a previous review. Of the 17 patients with a BMI over 30, none (0%) had been directed to explore Weight Management tools beyond diet and exercise advice.

**Conclusion:** Patients under the care of secondary psychosis services were not advised to discuss further weight management options with their GP. Highlighting a vital missed opportunity to provide care that could have long-term impacts on patients.

This leaves us with the vital question: Could we do more to advocate for this patient group, who may not have the financial or social capital to seek the management that they are entitled to?

